# Trigeminal neuralgia caused by a persistent primitive trigeminal artery variant passing through Meckel’s cavity: a case report

**DOI:** 10.1186/s12883-023-03483-1

**Published:** 2023-12-07

**Authors:** Zhengyan Zhu, Zhenpan Zhang, Risheng Liang

**Affiliations:** Department of Neurosurgery, Fujian Medical University Union Hospital, Fuzhou, Fujian Province China

**Keywords:** Anatomical variation, Microsurgical vascular decompression, Persistent primitive trigeminal artery variant, Trigeminal neuralgia

## Abstract

**Background:**

Persistent primitive trigeminal artery variant (PPTAv) is a rare remnant of the primitive intracranial embryonic anastomotic arteries, and its persistence has an unknown etiology. Trigeminal neuralgia attributed to a PPTAv passing through Meckel’s cavity is extremely uncommon.

**Case presentation:**

A 73-year-old woman presented with right-sided facial pain for 10 years that had failed to respond to medication. Magnetic resonance angiography suggested the presence of a PPTAv compressing the trigeminal nerve, as the abnormal artery originated from the right internal carotid artery. During microvascular decompression (MVD), the offending vessel was inferred to be a PPTAv, as it continued to become the anterior inferior cerebellar artery after passing through Meckel’s cavity. Postoperative computed tomography angiography showed the PPTAv continuing posteriorly as the anterior inferior cerebellar artery and supplying the cerebellar hemisphere, which confirmed the intraoperative judgment. The pain resolved after MVD and has not recurred in 12 months of follow-up.

**Conclusion:**

MVD is the best surgical choice for trigeminal neuralgia combined with a PPTAv. For patients with neurovascular conflicts, particularly those with suspected vascular variations, preoperative imaging examinations play a critical role in meticulously evaluating the anatomical locations of the nerves and blood vessels. Semilunar puncture (for radiofrequency ablation or percutaneous balloon compression) is contraindicated in patients with a PPTAv.

**Supplementary Information:**

The online version contains supplementary material available at 10.1186/s12883-023-03483-1.

## Background

A persistent primitive trigeminal artery (PPTA) is an embryonic anastomosis vessel between the internal carotid artery (ICA) and the basilar artery (BA); this rare cerebrovascular dysplasia can cause trigeminal neuralgia (TN). A PPTA variant (PPTAv) originates from the ICA and continues to become the cerebellar artery without connecting to the BA. We report a unique case of TN caused by a PPTAv that also passed through Meckel’s cavity.

## Case presentation

**Fig. 1:**
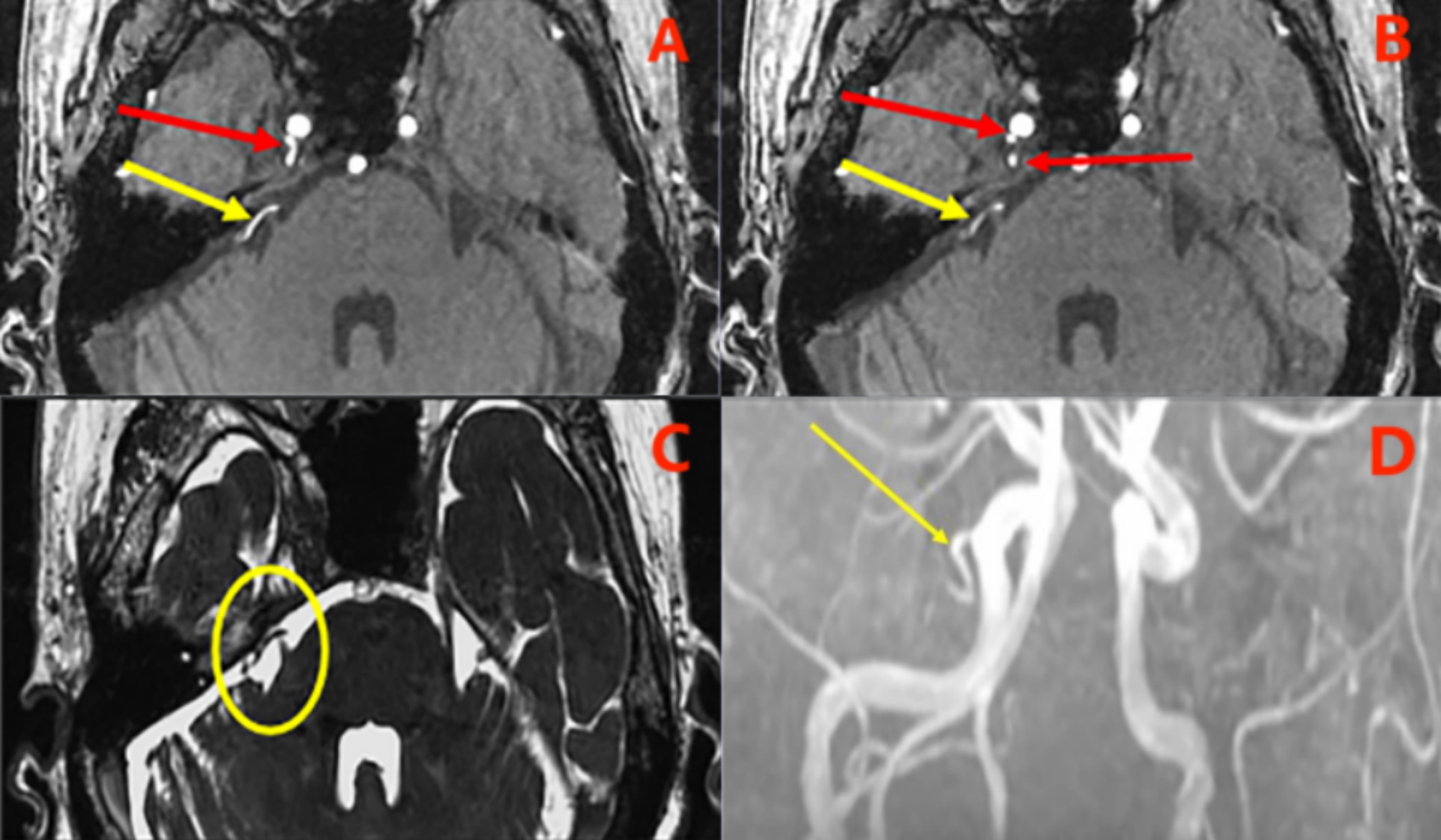
Preoperative magnetic resonance images. **A**&**B**, the original three-dimensional time-of-flight magnetic resonance angiography shows abnormal vessel compression in the cisternal segment of the right trigeminal nerve (yellow arrows). A persistent primitive trigeminal artery may originate from the right internal carotid artery (red arrows), which may enter Meckel’s cavity and be closely associated with the trigeminal nerve. **C**, three-dimensional fast imaging employing steady-state acquisition images show abnormal vessel compression in the cisternal segment of the right trigeminal nerve (yellow oval). **D**, preoperative maximum intensity projection reconstructed magnetic resonance angiography. A persistent primitive trigeminal artery (yellow arrow) originates from the precavernous portion of the right internal carotid artery

A 73-year-old woman was admitted to our hospital with severe pain on the right side of her face. Ten years ago, she was diagnosed with TN in another hospital and managed with carbamazepine. However, the efficacy of the medication waned as the disease progressed. Two months before the present admission, the pain had worsened and the medication had become completely ineffective. The patient had been diagnosed with hypertension 8 years ago, and her blood pressure on admission was 164/87 mmHg. Neurological examination revealed a trigger point on the right side of the face, with no other discernible abnormalities. Three-dimensional time-of-flight magnetic resonance angiography and fast imaging employing steady-state acquisition revealed compression of a small artery in the pontine cisterna segment of the right trigeminal nerve. The abnormal artery was suspected to be a PPTA that might originate from the precavernous portion of the right ICA (Fig. 1). After admission, the patient’s blood pressure was effectively managed.

**Fig. 2:**
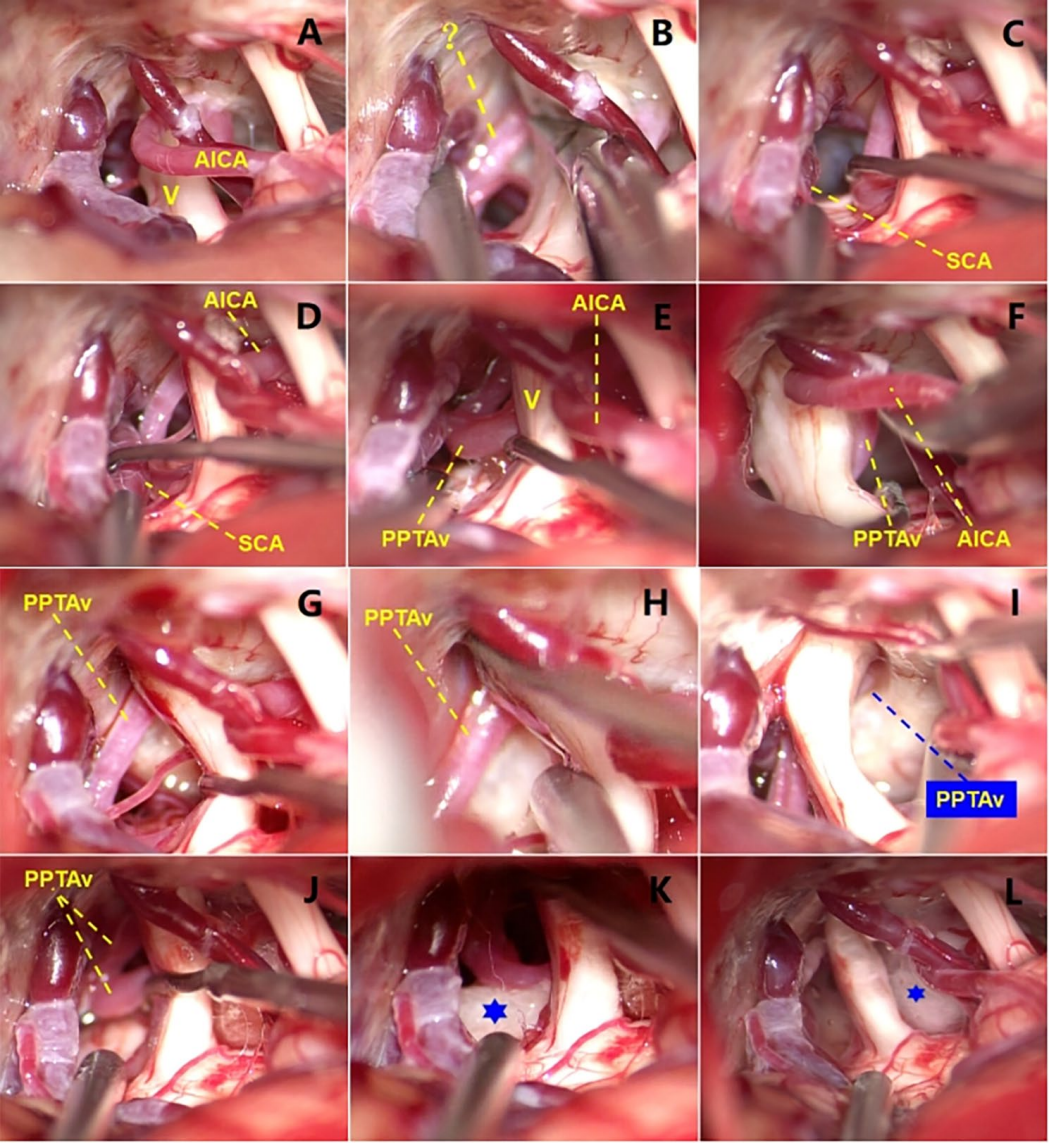
Intraoperative exploration and separation of the PPTAv and AICA. **A, B**, At initial view, the AICA was closely related to the V, and an artery of unknown source above the latter, possibly indicating the presence of a PPTAv. **C, D**, Exploring the tentorium edge, the SCA was found to have no contact with the V. **E, F**, In front of the root of the V, the unknown artery (?) continued as the AICA, twisting around and compressing the V, which led us to consider the unknown artery to be a PPTAv. **G-J**, Exploration of Meckel’s cavity confirmed that the unknown artery (?) was a PPTAv that passed Meckel’s cavity, forming a loop on the surface of the pons. **K, L**, Teflon felts (✡) were placed as separation agents between the V and PPTAv. PPTAv, persistent primitive trigeminal artery variant; AICA, anterior inferior cerebellar artery; V, trigeminal nerve; SCA, superior cerebellar artery

Following comprehensive discussions with the patient and her family regarding the surgical options and associated risks, microvascular decompression (MVD) was performed via the right suboccipital retrosigmoid approach. Intraoperatively, the neurovascular structures within the right cerebellopontine angle were meticulously dissected, and the close anatomical relationship between the anterior inferior cerebellar artery (AICA) and the trigeminal nerve was revealed. An unidentified artery was observed superior to the trigeminal nerve. No contact between the superior cerebellar artery (SCA) and the trigeminal nerve was found when exploring toward the tentorial margin. Subsequently, the culprit unidentified artery was found to pass through Meckel’s cavity and reach the surface of the pons. A concomitant relationship between the culprit artery and the trigeminal nerve in the direction of Meckel’s cavity was observed. The culprit artery then descended to form a loop in front of the root of the trigeminal nerve before curving posteriorly and continuing as the AICA; thus, it was identified as a PPTAv. The PPTAv was cautiously detached from the trigeminal nerve. Following this, mini-volume Teflon felts were gently positioned between the trigeminal nerve and the PPTAv–AICA without exerting force (as a separation barrier, while the limited local space and intricate course of the artery and vein made it difficult to perform slinging technique). Special attention was paid not to leave any blood on Teflon felts. (Fig. 2).

Postoperatively, the patient experienced complete resolution of symptoms without any complications. Postoperative computed tomography angiography showed the PPTAv continuing posteriorly as the AICA and supplying the cerebellar hemisphere, which confirmed the intraoperative judgment (Figure S1). Re-examination of the magnetic resonance angiography images revealed that the vascular compression in the cisternal segment of the right trigeminal nerve had disappeared and the right trigeminal nerve had become thicker (Figure S2). The patient was discharged from the hospital on postoperative day 9 and had no recurrence of pain in 12 months of follow-up.

## Discussion and conclusions

### Embryology and classification of PPTA

At the developmental stage of a 3-mm embryo, the trigeminal artery arises at the junction of the paired dorsal aorta and the first aortic arch. During the developmental phase of the embryo at 4 mm, the ICA supplies the longitudinal neural arteries (the future BA) via the trigeminal artery, internal auditory artery, and sublingual artery [1]. Subsequently, the embryonic anastomoses between the ICA and vertebrobasilar arteries usually degenerate. However, in cases of developmental anomalies, these arteries may persist into adulthood, forming embryonic residual blood vessels between the ICA and the vertebral BA, such as a PPTA [1]. Most PPTAs (85%) originate from the cavernous portion of the ICA, while some PPTAs (15%) arise from the petrous segment [2]. Moreover, as a congenital vascular anomaly, a PPTA may alter the hemodynamics of the circle of Willis, predisposing patients to arterial aneurysm formation. Ladner et al. [3] reported a case of an aneurysm associated with a PPTA.

On the basis of its positional relationship with the abducens nerve, a PPTA can be classified as lateral or medial. The medial type of PPTA originates from the posteromedial segment of the cavernous portion of the ICA and courses along the medial side of the abducens nerve under the pituitary fossa, piercing the dorsum sellae dura to anastomose with the BA. The lateral type of PPTA arises from the posterolateral segment of the cavernous portion of the ICA and courses along the lateral aspect of the abducens nerve, piercing the dura of the posterior cranial fossa medial to the sensory root of the trigeminal nerve and anastomosing with the BA [4].

Saltzman used the angiographic characteristics to classify PPTAs into three types. Saltzman type I PPTA supplies both the bilateral SCAs and posterior cerebral arteries, with the absence of an ipsilateral posterior communicating artery. Saltzman type II PPTA supplies the bilateral SCAs, while the posterior cerebral arteries are supplied by the posterior communicating arteries [5]. Saltzman type III PPTA arises from the cavernous portion of the ICA without anastomosing with the BA [6]. In our case, the preoperative imaging and intraoperative anatomical exploration revealed the PPTAv passing through Meckel’s cavity and continuing posteriorly as the AICA. Therefore, the PPTAv was classified as Saltzman type III, but with a peculiar anomaly of passing through Meckel’s cavity. To our knowledge, this is the first reported case of TN in nearly 30 years in which such a vascular variation has been confirmed with clear intraoperative imaging. New PPTA variants have also been reported in recent years and have yet to be included in the Saltzman classification. These cases involve the coexistence of two distinct vascular anomalies: PPTA associated with duplicate BA or fenestration of the cavernous portion of the ICA [7, 8].

### PPTA and TN

TN is usually caused by vascular compression of the trigeminal nerve. Congenital vascular anomalies can complicate anatomical relationships and increase the risk of TN. The prevalence of PPTA among patients with TN is 2.2%, which is significantly higher than the prevalence in the general population [1]. Some reports even indicate that the PPTA and its branches jointly compress the trigeminal nerve, contributing to the pathogenesis of TN [9]. Symptoms of TN can also be manifested in patients with coexisting PPTA and hypertension [10]. A PPTAv is more likely to cause TN than a PPTA [11]. Miki et al. [12] reported a case of TN caused by a PPTAv and the SCA. In our case, the PPTAv originated from the precavernous portion of the ICA and passed through Meckel’s cavity. Subsequently, the PPTAv reached the surface of the pons, then became entangled and compressed the trigeminal nerve and finally continued posteriorly as the AICA. The prevalence of a PPTAv is only 0.18% [13]. A review of the literature from 1990 to 2023 revealed only five cases of TN confirmed to be associated with a PPTAv (Table 1) [12, 14–17].

**Table 1:** Summary of published cases of trigeminal neuralgia caused by a persistent primitive trigeminal artery variant

Patient	Author/Year	Sex	Age(Y)	Laterality of TN	Neuroimaging diagnosis of PPTAv	Intraoperative diagnosis of PPTAv	PPTAv through the Meckel’s cavity	Course of PPTAv
1	Tokimura H et al.1990 [14]	M	48	Left	Post-op	N	N	PPTAv originates from the left ICA and continues as the left AICA
2	Kawahara I et al.2011 [15]	F	86	Right	N	Y	N	PPTAV originates from the right ICA and continues as the right AICA
3	Son B et al.2013 [16]	F	46	Left	Pre-op	No surgery	?	PPTAv originates from the left ICA and continues as the left AICA
4	Miki K et al.2019 [12]	M	65	Left	Pre-op	Y	?	PPTAv originates from the left ICA and located in the posterior fossa
5	Ling MM et al.2020 [17]	F	74	Left	Pre-op	No surgery	N	PPTAv originates from the left ICA and continues as the left AICA

AICA, anterior inferior cerebellar artery; ICA, internal carotid artery; PPTAv, persistent primitive trigeminal artery variant; Post-op, postoperative; Pre-op, preoperative; ?, neuroimaging revealed the passage of the PPTAv through Meckel’s cavity, but there was a lack of intraoperative anatomical evidence; TN, trigeminal neuralgia

### Treatment of TN

The gold standard treatment for TN is MVD [18], which results in immediate pain relief in 90–95% of patients [19]. Nascimento et al. [20] examined the outcomes after MVD and percutaneous balloon compression (PBC) procedures and concluded that MVD was the optimal surgical choice for TN. Di Carlo et al. [21] investigated the short- and long-term clinical outcomes of MVD for TN, substantiating its safety, efficacy, and low rate of severe postoperative complications. The present study emphasized that the long-term outcomes of TN are associated with the type of vascular structure involved.

Careful preoperative neuroimaging assessments are essential to choose the best surgical approach, especially when a vascular anomaly is suspected. Surgeons should be aware of the possibility of various vascular anatomical variations, such as a PPTA. Furthermore, precise intraoperative management is crucial in preventing injury to vital neurovascular structures and achieving satisfactory outcomes. In our case, preoperative radiological examinations showed that the TN might be caused by the PPTA compressing the trigeminal nerve. Intraoperative anatomical exploration revealed that the offending vessel passed through Meckel’s cavity and continued posteriorly as the AICA, which was diagnosed as a PPTAv. This culprit vessel compressed the trigeminal nerve in its cisternal segment. Postoperatively, the patient’s symptoms completely disappeared, while also hinting that the PPTAv did not compress the trigeminal nerve within the Meckel’s cavity.

Semilunar puncture (to perform PBC and radiofrequency ablation) may injure a PPTA or PPTAv passing through or close to Meckel’s cavity and lead to catastrophic hemorrhage. Consequently, the semilunar puncture procedure is contraindicated for patients with such anatomical anomalies, for whom MVD is the only surgical choice.

### Conclusion

We report a unique case of TN attributed to a PPTAv that passed through Meckel’s cavity and continued as the ipsilateral AICA, compressing the trigeminal nerve. For patients with neurovascular conflict, meticulous preoperative assessments and standardized intraoperative management are essential to achieve optimal surgical outcomes. MVD is the best surgical choice for patients with vascular anatomical variations similar to the present patient, while semilunar puncture (to perform radiofrequency ablation or PBC) is contraindicated.

Please refer to the supplementary materials for detailed postoperative images.

### Electronic supplementary material

Below is the link to the electronic supplementary material.

Supplementary Material 1

## Data Availability

All data generated during the project will be made freely available upon reasonable request. There are no security, licensing, or ethical issues related to these data.

## Abbreviations

PPTAv: Persistent primitive trigeminal artery variant
PPTA: Persistent primitive trigeminal artery
MVD: Microvascular decompression
AICA: Anterior inferior cerebellar artery
ICA: Internal carotid artery
BA: Basilar artery
TN: Trigeminal neuralgia
SCA: Superior cerebellar artery
