# Supplementary material for: Trigeminal neuralgia caused by a persistent primitive trigeminal artery variant passing through Meckel’s cavity: a case report
Source: BMC Neurol. 2023 Dec 7;23:432. doi: 10.1186/s12883-023-03483-1 (PMC10702054; doi:10.1186/s12883-023-03483-1)
Supplement: Supplementary file 1 — Supplementary Material 1 [file 12883_2023_3483_MOESM1_ESM.docx]

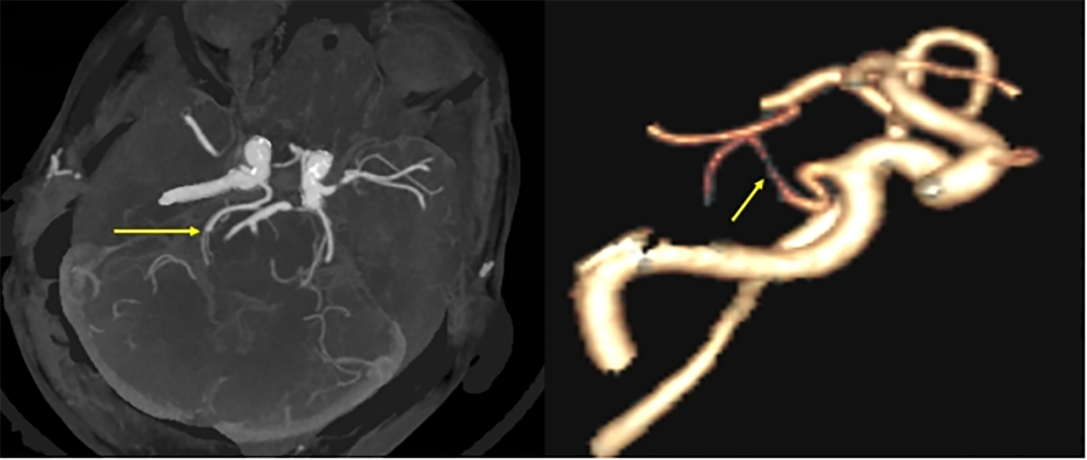


Figure S1 Postoperative computed tomography angiography. The persistent primitive trigeminal artery variant (yellow arrows) extends posteriorly into the anterior inferior cerebellar artery and supplies the cerebellar hemisphere.


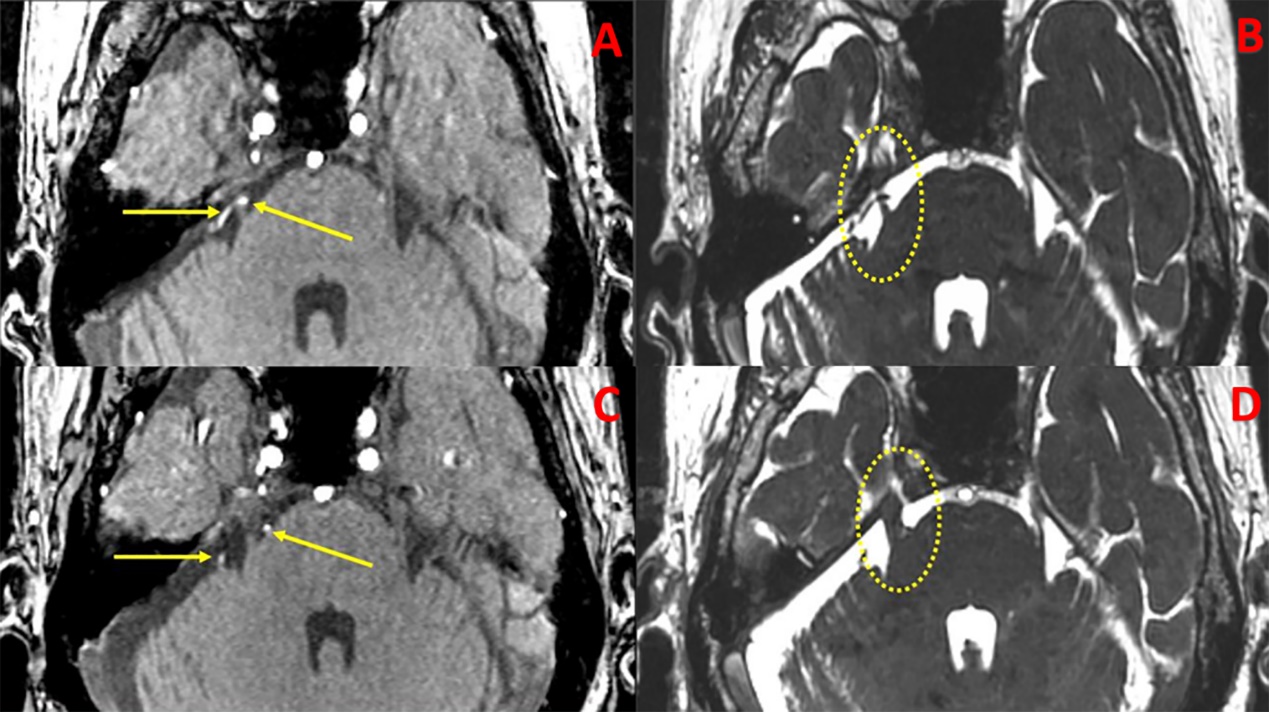


Figure S2 Comparison of magnetic resonance images. A&B, images obtained before surgery. C&D, images obtained after surgery. Re-examination of magnetic resonance angiography images postoperatively revealed the disappearance of vascular compression (yellow arrows) in the cisternal segment of the right trigeminal nerve, and the right trigeminal nerve (yellow ovals) became thicker.
